# Supplementary figures and images for: Individual behavioral type captured by a Bayesian model comparison of cap making by sponge crabs
Source: PeerJ. 2020 May 14;8:e9036. doi: 10.7717/peerj.9036 (PMC7231507; doi:10.7717/peerj.9036)

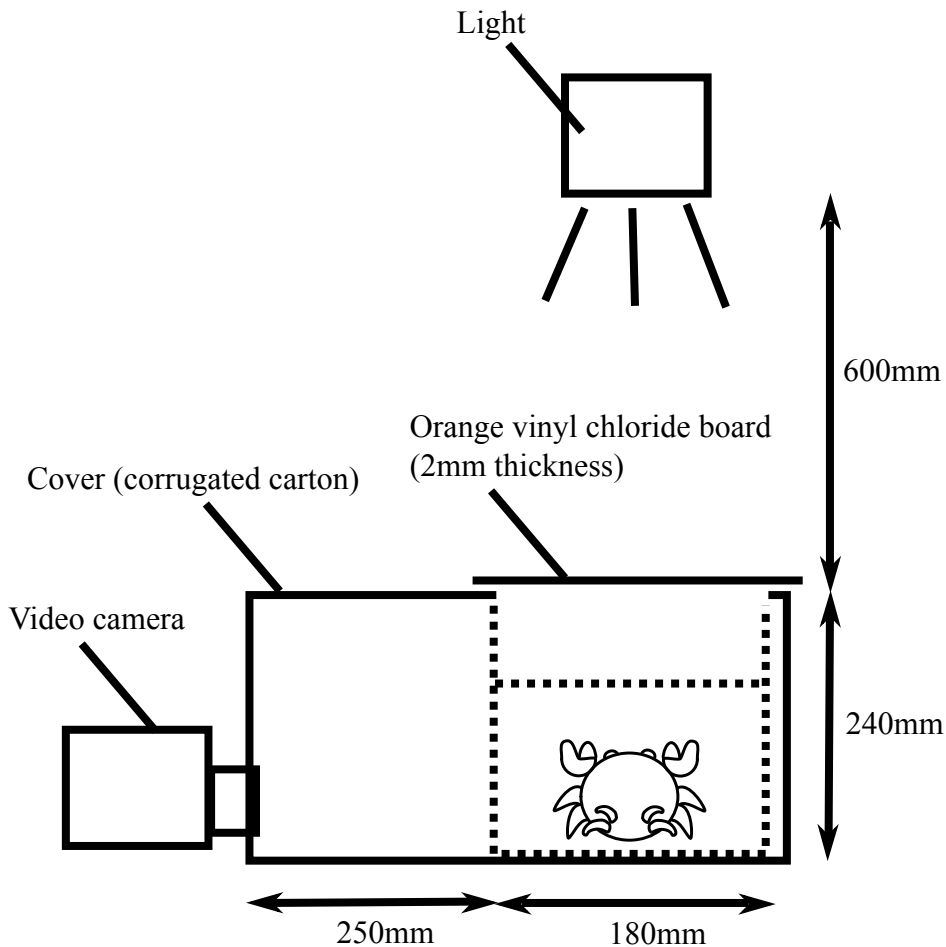

Supplement: Figure S1 — The cap making and carrying behavior was video recorded in this setup. [file peerj-08-9036-s001.pdf]
